# The effect of telephone counseling and internet-based support on pain and recovery after tonsil surgery in children – a systematic review

**DOI:** 10.1016/j.ijnsa.2021.100027

**Published:** 2021-04-22

**Authors:** Gunnhildur Gudnadottir, Rebecca Gagnemo Persson, Eva Drevenhorn, Eva Olofsson, Helena Rosén

**Affiliations:** a Department of Otolaryngology, Sahlgrenska University Hospital, Sahlgrenska Academy, Gothenburg University, Gothenburg, Sweden; b Department of Health Sciences, Faculty of Medicine, Lund University, Lund, Sweden; c Department of Paediatric and Adolescent Medical Care, Skaraborg Hospital, Skövde, Sweden

**Keywords:** Telephone counselling, Internet-based support, Pain, Recovery, Tonsil surgery, Children systematic review

## Abstract

**Objectives:**

The recovery after tonsil surgery is often troublesome for children and caregivers often feel insecure regarding optimal post-operative care for their children at home.

The aim was to study what the current literature reports regarding the effect of post-operative telephone counselling and Internet support on pain and recovery after paediatric tonsil surgery.

**Method:**

A systematic literature review was conducted where only randomised clinical trials were included.

**Outcome measures:**

Primary outcome measure was pain after surgery. Secondary outcomes also included nausea, anxiety, children's knowledge, use of analgesics, fluid intake and health care service use.

**Results:**

Only four studies fulfilled the inclusion criteria. The studies were heterogeneous, rendering a meta-analysis impossible. The results of the included studies showed a possible positive effect on postoperative pain, as well as level of anxiety, use of analgesics, fluid intake and health care service use. However, the studies were few with few included participants.

**Conclusion:**

There were indications, but no definitive evidence supporting the positive effect of telephone counselling or Internet-based support on pain and recovery after tonsil surgery in children. More research is needed to further examine these effects.

ClinicalTrials.gov 12/03/2017 (NCT03292068).

**What is already known about the topic**

• Tonsil surgery is known to cause more postoperative problems than many other types of surgery.
• Different interventions have been implemented to help improve parental management of their children´s postoperative pain at home.
• Earlier interventions showed modest improvements.

**What this paper adds**

• Perioperative benefits worth mentioning that emerged in this review were pre-surgical preparations and nurse telephone follow up might positively influence children's knowledge, level of anxiety, use of analgesics, perceived pain, fluid intake and health care service use.
• More studies that match the inclusion criteria and having the same type of intervention design are still needed to further examine these effects.

## 1 Introduction

Tonsil surgeries are among the most frequently performed surgical procedures on children in general anesthesia. The most common indications for tonsil surgery in children are obstructive breathing and chronic tonsillitis. The postoperative period can often be troublesome due to pain, nausea, dehydration and general malaise (Hamers and Abu‐Saad, 2002; Stanko et al., 2013; Sutters and Miaskowski, 1997). Previous research has shown that parents are insecure regarding how to handle their children´s pain after surgery and ensuring adequate fluid intake even though they receive extensive information before surgery (Bartley and Connew, 1994; Longard et al., 2016; Stanko et al., 2013). This may result in inadequate pain management causing unnecessary suffering for the children as well as unplanned health care contacts that could possibly be avoided with proper support after surgery (Rungby et al., 1999). A significant number of children have acute revisits after tonsillectomy (Edmonson et al., 2015; Mahant et al., 2014; Shay et al., 2015). Tumin et al., (2017) found that 15% of 16 608 children returned to the hospital within 30 days following surgery and Curtis et al., (2015) found similar numbers, 13.3% of 3 198 (mean time 4.8 days postoperative). Reported reasons for acute revisits include poor analgesia use, fever, dehydration and nausea (Curtis et al., 2015; Edmonson et al., 2015; Shay et al., 2015). Since the majority of these tonsillectomy procedures are performed as day surgery, with the patient returning to home on the day of surgery, it may be challenging to ensure that the parents receive proper information and support. Nurse contact via telephone and/or Internet may be an interesting and cost-effective option for these parents and their children (Erskine et al., 2015; Lauder & Emmott, 2014; Sutters et al., 2005).

Different interventions have been implemented to help improve parental management of their children´s postoperative pain at home. The choice of analgesics is of large importance, as is the timing and amount of analgesics given (Erskine et al., 2015; Lauder and Emmott, 2014; Sutters et al., 2005). This has been extensively studied. Another important aspect, that is not as well studied, is the effect of information and support to the parents as well as the child on pain and recovery after surgery. This may be accomplished by various methods, such as regular nurse follow-up by telephone or home visits as well as text message or online-information in the pre- and postoperative period and increased preoperative education regarding how to handle pain. A systematic review was conducted in 2014 to assess interventions aimed at improving parents’ management of their children's postoperative pain at home such as pain education, training in pain assessment, education on distraction, instruction in around-the-clock dosing and nurse coaching (Chorney et al., 2014). The included methods showed modest improvements. The study included different types of surgeries. Since tonsil surgery is known to cause more postoperative problems than many other types of surgery (Williams et al., 2015) it is of interest to examine the effects on tonsil surgery specifically.

The aim was to study what the current literature reports on the effect of post-operative telephone counselling and Internet support on pain and recovery after paediatric tonsil surgery.

## 2 Material and methods

The choice of search words was based on the PEO framework (Population, Exposure, Outcome). Keywords were chosen as follows: children (Population), telephone or Internet follow-up after tonsil surgery (Exposure), pain and recovery (Outcome). Prior to the study, a search for earlier literature reviews was conducted in the Cochrane library but none were found. A search for intervention studies was made in April 2018, and updated in February 2019, in the databases Medline (PubMed) and Medline (Embase) using the MeSH-terms tonsillectomy/tonsillotomy, telenursing/telephone (Table 1) and in CINAHL using the subheadings tonsillectomy/tonsillotomy and web/online/telephone/telenursing/Internet/after care/postoperative care. The included studies were reviewed following a checklist for assessing the quality of randomized studies (SwedishAgency for Health Technology Assessment and Assessment of Social Services). Exclusion criteria were pilot studies and studies not being randomized controlled trials, or not written in English.

**Table 1:** Search strategy in the database Medline (PubMed) in April 2018.

MeSH-terms	Results (no of papers)
#1tonsillotomy OR tonsillectomy	11 113
#2web* OR online OR telephone OR telenursing OR internet OR "after care" OR(MH "Postoperative Care")	445 009
#3#1 AND #2	480
#4#3 AND Limiters - Published Date: 19980101-20181231Language: English	271

Duplicates were excluded, and the remaining titles and abstracts were read and selected independently by all the authors. In the next step the authors selected what studies were to be read in full text and those who met the eligibility criteria were included. In case of divergence between the authors, the inclusion or exclusion of the papers was decided through discussion until consensus was reached. The included studies were reviewed following a checklist for assessing the quality of randomized studies (Swedish Agency for Health Technology Assessment and Assessment of Social Services). The checklist covers risk of systematic errors and the risk of conflict of interest. In the assessment of systematic errors are a study's selection bias, performance bias, detection bias, attrition bias and reporting bias included. The risk assessment was made by answering yes, no, unclear or not applicable to a number of questions for each bias area (Table 2).

**Table 2:** Studies on telephone and Internet follow-up after paediatric tonsil surgery included in the review.

Author/Year/Location	Study Design	SB	PB	DB	AB	Sponsors/doners	Participants	Intervention and Control Groups	Outcome measures	Measurement used	Results
Xin et al., 2018China	RCT	+	NA	+	++	no	4-12yIG *n*=382 CG *n*=389	IG: nurse telephone follow-up POD 1,3,7,14CG: doctors’ visits at hospital POD 1,3,7,14	Pain, postoperative complications, analgesics administered, use of healthcare services	*Pain*: The Wong-Baker Faces Pain Scale	IG: Less pain, more analgesics, improved fluid intake POD 1+3, more vomiting POD 1, lower health care service use.
Yang et al., 2016Korea	RCT	+	NA	++	++	no	3-7yIG *n*=27CG *n*=34	IG: 10 text messages: on the day of hospitalization; on the day of the operation; on the day of discharge; every day from discharge to attendance at the outpatient clinic. Conventional text and verbal informationCG: conventional text and verbal information	Mothers knowledge score: the day of hospitalization and POD 7Children's anxiety level: on the day of hospitalization, just before operation. Sick-role behavior: POD 7	*Mothers’ knowledge:* a 16-item scored questionnairedeveloped for this study. *Children's anxiety:* Amodified version of an eight-item self-report instrument. *Children's Sick-role behavior:* A questionnaire comprising nine items with a 3-point Likert scale	IG: Mothers had higher knowledge scores.IG: Children were less anxiousDifference in sick-role behavior where children in IG received higher scores in three in nine categories
Paquette et al., 2013Canada	RCT	+	NA	+	+	no	4-12yIG *n*=24CG *n*=21	IG: Nurse telephone follow-upPOD 1,3,5 and 10CG: standard postoperative instructions by a nurse.	Pain intensity,analgesicsadministered, presenceof postoperativecomplications, use ofhealthcare services	*Pain*: Revised Bieri Faces Pain Scale (FPS-R) (4-7y) and a numerical rating scale from 0–10 (8-12Y)	IG: Received more analgesics increased fluid intake POD 1 + 3, more constipation at POD 3.No difference regarding pain intensity or use of healthcare resources.
O'Conner- Von et al., 2008USA	RCT	++	NA	+++	++	no	10-16 yIG n=28CG n=14NT n=24	IG: Internet preparation program viewed at home before surgeryCG: Standard preparation program in the hospital setting before the day of surgeryNT: The routine preparation program on the day of surgery	Anxiety, knowledge score, pain intensity,satisfaction with method	*Anxiety:* State-Trait Anxiety Inventory for Children (STAIC), State-Trait Anxiety Inventory (STAI).*Knowledge*: The Knowledge Questionnaire*Pain*: A pain intensity scale.*Satisfaction*: The Adolescent and Parent Satisfaction Questionnaire.	IG: Better knowledge and higher satisfaction with the methodNo difference regarding anxiety and pain intensity.No difference inpreoperative anxiety forthe parents

Categories for risk of bias: + low risk of bias; ++ medium risk of bias; +++ high risk of bias. Abbreviations: NA=not applicable.

SB= Selection bias, PB= Performance bias, DB= Detection bias, AB= Attrition bias, RB= Reporting bias,

C= Conflict of interests, IG=Intervention group, CG=Control group, NT=Non-treatment group, POD=Post-operative day

## 3 Results

The search in Medline gave 271 studies and CINAHL 112 studies. Altogether 329 studies, when duplicates were removed. The updated search in February 2019 in the same databases and with the same search terms, resulted in 16 new studies.

**Fig. 1:**
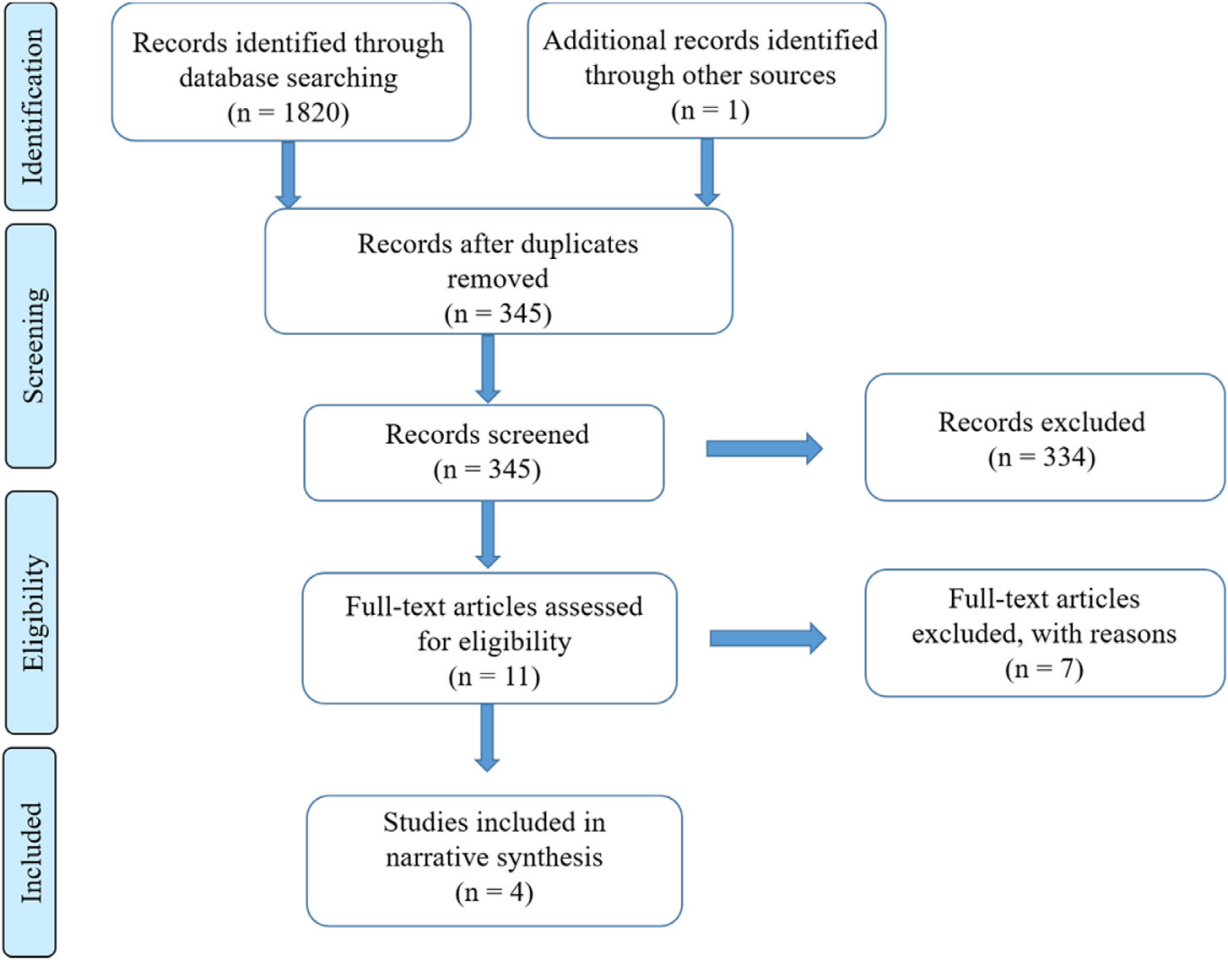
Study Flow Diagram according to PRISMA. Reasons for exclusion on the eligibility level were that the articles did not fulfil the inclusion criteria; two were pilot studies, one was studying parents’ adherence to postoperative diet and physical activity instructions and four were studying parents’ involvement in the postoperative care at home in different ways.

After reading all 345 abstracts, 11 studies were at first assessed as relevant to be included in the review by any of the authors. After reading the 11 papers only four studies were found to fulfil the aim of the literature review. The included studies are presented in Table 2. Seven articles were excluded. These can be seen in other material. Reasons for exclusion were that they did not fulfil the inclusion criteria; two were pilot studies, one was studying parents’ adherence to postoperative diet and physical activity instructions and four were studying parents’ involvement in the postoperative care at home in different ways, see Flow Diagram according to PRISMA, Preferred Reporting Items for Systematic Review and Meta-analysis (Moher et al., 2010) (Fig. 1).

The included papers were studies on non-pharmacological interventions on pain and recovery after tonsillectomy in children such as telephone follow-up, education using short message service and education using an Internet program. Assessment of the risk of bias in these studies is presented in Table 2.

### 3.1 Telephone follow-up

Two of the randomized clinical trials, included in the study were aimed at determining if a nurse telephone follow-up could substitute clinic visits, and decrease pain intensity, incidence of postoperative complications, and additional healthcare resource use in paediatric tonsillectomy (4–12 years) (Paquette et al., 2013; Xin et al., 2018). Pain intensity was used as the primary outcome.

Xin et al., (2018) found that nurse telephone follow-up on postoperative day 1, 3, 7 and 14 significantly reduced pain intensity, promoted analgesia use and fluid intake and reduced the seek for healthcare services when compared to visits to a doctor on the same days. The mean pain score using Scale ranging from 0 to 5 (Wong and Baker, 1988) on postoperative day 1 and 3 were significantly higher in the control group (*n* = 329) (postoperative day 1: 4.7 ± 1.1; postoperative day 3: 3.5 ± 1.2) compared with the intervention group (*n* = 341) (postoperative day 1: 4.2 ± 1.2; postoperative day 3: 2.7 ± 1.1). All of the patients in the intervention group and control group received some kind of follow up; either a nurse telephone follow up or a visit to the doctor at the hospital. There was no control group that did not receive any follow-up or with patients receiving standard care. In China, most children revisit the surgeons one week and one month after surgery.

In contrast (Paquette et al., 2013) did not find any significant differences regarding pain intensity or use of healthcare resources in a study that compared nurse telephone follow-up on postoperative day 1, 3, 5 and 10 to children that only received standard care with no follow-up. The intervention group (*n* = 24) received more analgesics on postoperative day 1 and 3, increased their fluid intake on postoperative day 1 and 3, but had more constipation on postoperative day 3 than the children in the control group (*n* = 21). There were no significant differences in pain intensity scores, when using the revised Bieri Faces Pain Scale (FPS-R) (4–7 years) (Hicks et al., 2001) and the Numerical rating scale, NRS (8-12 years) (Jensen et al., 1986), both ranging from 0 to 10. Also, there were no significant differences in use of health care resources, nausea or fever between the groups but it was pointed out by the study's authors (Paquette et al., 2013) that possibly the sample size was too small to detect any differences.

### 3.2 Education using smartphone text messages

Yang et al., (2016) conducted a randomized controlled trial to examine the effects of tonsillectomy education using smartphone text messages on mothers’ knowledge using a 16 item questionnaire developed for the study, on patient care and children's (3–7 years of age) anxiety using the Children's anxiety scale developed by Cho (2001), as a behavioural checklist to measure acute pain and anxiety and sick-role behaviour. Mothers who received information using smartphone text messages (*n* = 27) showed significantly higher knowledge scores than the mothers in the control group (*n* = 34) who received information by conventional textual and verbal means only. The children's anxiety increased in both groups but the children in the intervention group were less anxious than those in the control group. The children's sick-role behaviour also differed significantly between the two groups as the children in the intervention group, who were provided information by text messages received higher scores for ‘ice pack application’, ‘chewing gum’, and ‘eating ice cream in response to pain’ than the children in the control group. The number of messages sent and received was found to have been three times higher than planned which suggests that this may be an effective way for the parents to communicate with healthcare personnel regarding their children's recovery. The authors concluded that providing information by smartphone text messages helped to increase the knowledge of mothers’ of children who have had a tonsillectomy to relieve anxiety and to improve the child's sick-role behaviour.

### 3.3 Education using an internet program

O'Conner-Von (2008) compared the effectiveness of an Internet-based method with a standard method for preparing adolescent patients (defined in the article as children being between 10 and 16 years of age) scheduled for outpatient tonsillectomy procedures. The Internet preparation program was designed by the investigators and consisted of a description of the routines during outpatient surgery for the adolescents (intervention group *n* = 28), including recommendations for home care for their parents. The standard preparation program was offered in the hospital setting (control group *n* = 14). The adolescents that were assigned the intervention group initially but did not follow through with the protocol were assigned to a third, no treatment group (*n* = 24)) group after the start date of their participation in the study. A registered nurse and a child life specialist presented developmentally appropriate sensory and procedural information about the routines during surgery. A tour was offered for the adolescent and his or her parents, along with recommendations for home care. The outcomes measured were level of anxiety using the instruments State-Trait Anxiety Inventory for Children (STAIC) Spielberger and Rickman (1990) and State-Trait Anxiety Inventory (STAI) for adults Spielberger (1983), knowledge acquisition using The Knowledge Questionnaire (Siaw et al., 1986), pain using the NRS instrument (0-10), and satisfaction using The Adolescent and Parent Satisfaction Questionnaire, developed for the study, with the method of preparation, and parental level of anxiety and satisfaction with the method of preparation included.

There was a significantly increased knowledge acquisition (*p* = 0.001) and satisfaction (*p* = 0.004) among the adolescents in the intervention group. There was no difference in preoperative anxiety between the groups, neither for the adolescents nor their parents. Postoperative pain scores did not differ between the groups; however, the results did show a strong trend of higher pain scores across the groups with the intervention group having the lowest pain score and the non treatment group, having the highest pain score. The parents whose adolescents participated in the intervention group demonstrated a significantly (*p* = 0.004) higher level of satisfaction with the method of preparation for surgery. The authors propose that their Internet program for pre surgical preparation and other forms of health care education was particularly relevant for the adolescent population because of the unique characteristics associated with this stage of growth and development.

## 4 Discussion

This study has contributed a summary of the current state of knowledge within the topic of telephone counselling (Paquette et al., 2013; Xin et al., 2018), short message service (Yang et al., 2016) and Internet-based support (O'Conner-Von, 2008) on pain and recovery after tonsil surgery in children. The studies included in this review have been conducted in North America and Asia, which due to cultural differences may have had an impact on the outcome. For example, the children being followed up every week with doctor visits (Xin et al., 2018) may not be relevant in all countries.

The strengths of this study include the use of a systematic approach to search data from relevant databases, revealing most available papers on the subject i.e. effect of telephone counselling and Internet-based support. Furthermore, in consensus with AMSTAR, Assessing the Methodological Quality of Systematic Reviews (Shea et al., 2007), the authors of this study conducted the systematic review according to basic quality requirements. A list of included assessed studies are presented in our review (Table 2). The exclusion criteria guide the reader and contribute to the opportunity for replication of the study.

The main limitation of this review is the small number of studies that were assessed as suitable to be included, but also the heterogeneity of the included studies (such as the use of different instruments in measuring the same symptoms), well known as problematic in a review survey (Kontopantelis et al., 2013). We also found weaknesses/biases in all four included studies (Table 2). The included studies were few and heterogeneous and so a meta-analysis of the results was not possible. Instead the results for each study are described. Seven full text articles were excluded after being assessed (Anderson et al., 2017; Huth, Broome, & Good, 2004; Khan et al., 2017; Le, Drolet, Parayno, Rosmus, & Castiglione, 2007; Unsworth, Franck, & Choonara, 2007; Wiggins, 2009; Zagólski, O. et al 2010), (Table 3).

**Table 3:** Extended materials. Full text articles excluded after being assessed for eligibility. Abbreviation: RCT=Randomized Controlled Trial.

Author/Year/Location	Title	Study design	Outcome measures	Risk of bias	Reason for exclusion
Huth et al, 2004USA	Imagery reduces children's post-operative pain.	RCT7-12 yrsIG *n* = 38CG *n* = 371–4 h PO, 22–24 h PO	Pain, anxiety, medication	LowNo study protocol	The intervention is not within the inclusion criteria
Le, T. et al, 2007	Follow-up phone calls after pediatric ambulatory surgery for tonsillectomy: what can we learn from families?	Observation	Parents view Satisfaction with telephone call	High risk of bias.	The intervention is not within the inclusion criteria and high risk of bias. No RCT-study
Khan, S. et al, 2017	Utilization of a postoperative adenotonsillectomy teaching video: A pilot study.	RCTPilot study	Parents understanding and satisfaction.Analgesics.Child discomfort. Bleeding	Medium high	The intervention is not within the inclusion criteria
Anderson, M.E. et al, 2017	Preferred parental method of postoperative tonsillectomy and adenoidectomy follow-up (phone call vs. clinic visit).	Observation	Parents view	Medium high	The intervention is not within the inclusion criteria
Zagólski, O. et al 2010	Do diet and activity restrictions influence recovery after adenoidectomy and partial tonsillectomy?	Observation	Compliance to instructions.	High	The intervention is not within the inclusion criteria
Wiggins, S.A. et al, 2009	Family exemplars during implementation of a home pain management intervention.	Pilot study RCT + qvalitative	Around-the clock medical alarms	Medium high	The intervention is not within the inclusion criteria
Unsworth, V. et al 2007	Parental assessment and management of children's postoperative pain: a randomized clinical trial.	RCT	Pain. Number of days with analgesics and number of doses	Medium	The intervention is not within the inclusion criteria

As the postoperative period often is troublesome due to e.g. pain and nausea, interventions such as nurse telephone counselling follow-up could be beneficial to reduce the revisit rate with not only patient but also health economic profits. In the study of Xin et al., (2018) telephone follow up was compared with doctor's visit on the corresponding postoperative days. Interestingly, in the nurse follow-up group the children had less pain, used more analgesics and their fluid intake improved more than was found in the group that revisited their physician.

Despite mentioned limitations and research bias in the studies, some cautious conclusions can be made about nurse telephone follow up (Paquette et al., 2013; Xin et al., 2018) such as the children in the intervention group were given more analgesics and that their fluid intake increased. In Xin et al., (2018) the children in the intervention group also experienced less pain, and a lower level of health care service was used. There is a gap however in the knowledge whether nurse telephone follow up is a feasible intervention for children, parents and clinicians, to be included in routine follow-up care and thus likely to be satisfying for families. Furthermore, the impact on postoperative pain is unsure. Based on the existing evidence, nurse telephone follow-up post tonsillectomy is probably a beneficial method of postoperative management for paediatric patients, an effective intervention to help parents with pain management and appropriate administration of analgesics. However, more high-quality research is needed, preferably randomized controlled trials on larger patient groups to confirm these positive results.

Not only pain, but also anxiety is a common problem postoperatively as patients/parents report high levels of anxiety prior to and after tonsillectomy (Erkılıc et al., 2016). The result in Yang et al., (2016), where the children in the group that received text messages were less anxious than those in the control group, is supported by a study where they conducted a quality improvement project to develop a text messaging service to deliver messages to paediatric tonsillectomy patients to improve communication and overall experience Newton and Sulman (2016). They found that the intervention seemed to reduce anxiety and level of worrying during and after surgery. Since this study was not a randomized controlled trial, it was not included in this systematic review.

Not only postoperative communication but also interventions such as pre-operative booklets, smartphone app, text-message programs, video programs and Internet resource as well as caregiver programs may improve pain management and patient anxiety, and result in less emergency department visits (Levin et al., 2019). O'Conner-Von (2008) found that the children, who received the Internet preparation, had better knowledge and higher satisfaction and also showed the lowest pain score, however not significant. One major methodological weakness of the study was, however, that patients that did not follow through with the study protocol for the group they were randomised to, where then assigned to a non-treatment group. This reduces the reliability of their results.

In summary, some perioperative benefits worth mentioning that emerged in this review was that interventions like pre-surgical preparations (O'Conner-Von, 2008) and nurse telephone follow up (Paquette et al., 2013; Xin et al., 2018) might positively influence children's knowledge, level of anxiety, use of analgesics, perceived pain, fluid intake and health care service use. As already stated, due to the few studies within the topic of nurse telephone counselling and Internet-based support, no generalization can be made from our review. We can merely state that more studies that match the inclusion criteria and having the same type of intervention design are needed.

## 5 Conclusion

Taking the scientific quality of the included studies into account, we found indications, but no conclusive evidence about the effect of telephone counselling or Internet-based support on pain and recovery after tonsil surgery in children. There is a lack of well-designed studies and therefore recommendations for clinical counselling or support cannot be given. Thus, further well designed RTC studies are needed.

Implications for Nursing practice and science

Nursing interventions such as pre-surgical preparations and nurse telephone follow up may have a positive influence on children's postoperative recovery. This study also shows the need of further nursing studies within the area in order to be able to generalise results and thus draw evidence based conclusions.

## Declaration of Competing Interest

The authors declare that they have no competing interests.
